# Approaching Highly Leaching-Resistant Fire-Retardant Wood by In Situ Polymerization with Melamine Formaldehyde Resin

**DOI:** 10.1021/acsomega.1c01044

**Published:** 2021-05-06

**Authors:** Chia-feng Lin, Olov Karlsson, Jozef Martinka, Peter Rantuch, Edita Garskaite, George I. Mantanis, Dennis Jones, Dick Sandberg

**Affiliations:** † Wood Science and Engineering, Department of Engineering Sciences and Mathematics, Luleå University of Technology, Forskargatan 1, SE-931 77 Skellefteå, Sweden; ‡ Faculty of Materials Science and Technology, Slovak University of Technology, Vazovova 5, SK-811 07 Bratislava, Slovakia; § Lab of Wood Science and Technology, University of Thessaly, Griva 11, GR-43100 Karditsa, Greece; ∥ Department of Wood Processing and Biomaterials, Faculty of Forestry and Wood Sciences, Czech University of Life Sciences Prague, Kamýcḱ 1176, Praha 6 - Suchdol CZ-16521, Czech Republic

## Abstract

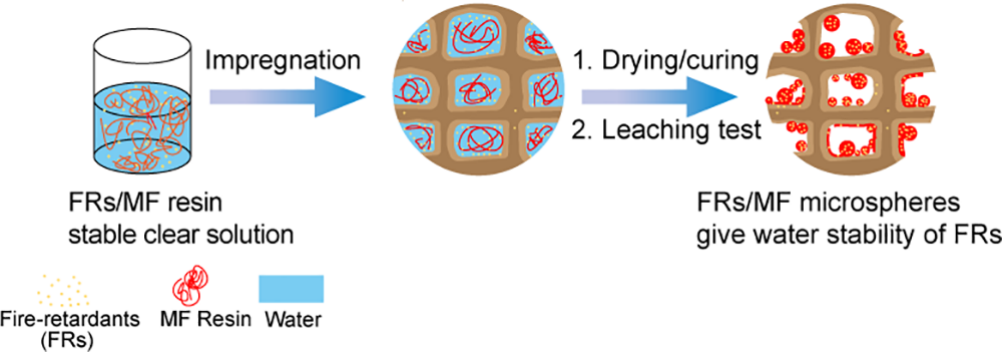

The objective of the work was to improve the leaching resistance of fire-retardant (FR) modified wood by the incorporation of a thermoset resin. Here, Scots pine (*Pinus sylvestris* L.) sapwood was impregnated with melamine formaldehyde (MF) resin and hydrophilic FRs guanyl-urea phosphate/boric acid by a vacuum-pressure treatment. Resistance to leaching of FR-modified wood was evaluated, after conducting an accelerated aging test according to European standard EN 84. Inductively coupled plasma analysis showed that the incorporation of MF resin significantly reduced the leachability of FRs. Scanning electron microscopy/energy-dispersive X-ray spectrometry revealed that the mechanism of water resistance was by doping the FRs into MF resin microspheres. Fourier transform infrared spectra showed the chemical functionality changes of FR-modified wood such as the formation of methylene bridges by drying the modified wood specimens. An increase in the thermal stability of FR-modified wood was confirmed by thermal gravimetric analysis. Excellent fire performance of FR-modified wood after leaching was affirmed by the limiting oxygen index and cone calorimeter tests.

## Introduction

Wood as a sustainable biopolymer has been widely used in a range of areas, including building construction panels and furniture. However, the inherent flammability of wood needs to be improved before subjecting it to advanced applications. Conventionally, the fire instability property can be improved by introducing salts containing nitrogen and phosphate ions and/or boron compounds into wood structure.^1−3^ The former could be di- and monoammonium phosphate, guanyl-urea phosphate (GUP), and melamine phosphate, whereas boric acid (BA) or borax is an example of the latter ones.^1,4−7^ The synergistic effect of nitrogen-phosphate salts and boron compounds provides a better fire-retardant property than their individual use.^8,9^ The fire-retardant mechanism of nitrogen-phosphate salts and boron compounds is believed to involve gas-phase and condensed-phase mechanisms.^1,10^ Nitrogen parts in nitrogen-phosphate salts are involved in the gas-phase mechanism by the release of noncombustible gases such as N_2_ or NH_3_ at elevated temperature, thereby diluting the contact to combustible gas O_2_ in the surroundings.^1^ The phosphate parts and boron compounds contribute to condensed-phase mechanisms by forming condensed protective inert layers, which prevent further thermal degradation. The inert layers from phosphates are due to phosphoanhydride bonds formed following dehydration of phosphate.^10^ Additionally, the phosphate polymers are effective when combined with materials having a high hydroxyl group content, such as wood, because they catalytically dehydrate wood to form char.^1,11^ Boron compounds dehydrate at relatively low temperatures and form protective glassy inert layers, which prevent the escape of flammable products, thereby hindering O_2_ from reaching the wood.^7^ Nevertheless, the majority of conventional fire retardants (FRs) are water-soluble. Thus, the treated wood products are mainly suitable for interior use, where exposure to liquid water is limited. They are not suitable for outdoor uses, although additional water-repellent protection layers have been applied on the surfaces of treated wood. These layers minimize moisture migration, which can eventually remove FRs during weathering exposure, which would ultimately lead to a product with reduced fire resistance.^12^ Therefore, the design of wood products for exterior purposes requires the fixation of the FRs within the wood structure.^13^

The fixation of the FRs within the wood structure can be achieved by two different approaches, namely, reactive- or composite-type fixation.^14^ Reactive-type fixation involves the formation of covalent bonds between FRs compounds and wood functionalities, such as the abundant hydroxyl groups in wood polymers, or by the stabilization of the FR compounds through further polymerization. For example, hydroxyl groups in the wood polymer have been esterified with organophosphorus and organoboron compounds.^15^ The ring-opening polymerization of the cyclic phosphorus monomer and the copolymerization of the organophosphorus monomers with poly(vinyl alcohol) or the methyl methacrylate monomer have been reported.^16−18^ Composite-type fixation occurs by mixing FRs into a polymer matrix so as to reduce the combustion behavior of the polymer matrix, e.g., blending ammonium dihydrogen phosphate into furfural alcohol for producing fire-retardant poly(furfural alcohol)-modified wood.^19,20^ Theoretically, in the reactive-type fixation, relatively low concentration of FRs can be used for achieving fire retardancy.^17^ However, the composite type dominates the market due to its easier preparation, by simply mixing conventional FRs with a polymer matrix without modifying the FRs’ formula.^21^ Thus, the composite type was chosen for this study.

The compatibility between the polymer matrix and FRs is important for composite-type fixation. As conventional FRs are water-soluble, a water-soluble MF prepolymer is chosen for better compatibility. Furthermore, the cured MF resin is colorless and hydrophobic, which is expected to provide good water resistance of the composite. The history of using MF resin-modified wood has been investigated since the 1930s.^22^ After impregnating the MF prepolymer into the wood structure, the cured polymer would reinforce the wood matrix and enhance properties such as dimensional stability, hardness, biological resistance, weathering resistance, and, to a certain extent, fire retardancy.^22−26^ MF resin-modified wood has been shown to have better fire retardancy than untreated wood; nevertheless, it is still inferior to the conventional nitrogen-phosphate salt/boron compounds used to modify wood due to the extra heat release during its combustion.^2,25^ Therefore, FRs were suggested to be blended in MF resin to improve fire stability, i.e., mixing phosphoric acid and/or boron compounds to MF resin before the treatment.^27−29^ Nevertheless, the applications are mainly limited to surface treatment or producing woodchip-based products due to the accelerated polymerization of MF resin caused by the acidity of FRs.^27,28^

The purpose of the work is to explore the possibility of upgrading FRs applied for interior-use to exterior-use application by the incorporation of MF resin. Here, we use a low-viscosity MF prepolymer suitable for preparation of the FR formulation to be impregnated into Scots pine (*Pinus sylvestris* L.) sapwood. GUP/BA was chosen as FRs due to commercial availability and fire-retardant efficiency. MF resin is expected to encapsulate the hydrophilic FRs by an organic sol–gel doping mechanism to improve the doped FR water resistance.^19,30^ The leaching resistance of the FRs was evaluated using the EN 84 European standard for accelerated aging tests, through repeatedly soaking the specimens in excessive amounts of water.

## Results and Discussion

### Formulation Viscosity and Curing Conditions

The viscosity of the formulation before and after impregnation was compared to determine the stability of the formulation. The spontaneous step-growth polymerization of the MF prepolymer would increase the viscosity over time and thus inhibit the penetration into the wood structure. Table 1 shows the viscosity of the formulations of 0-30MF (30 wt % MF resin) and 8-30MF (8 wt % FRs and 30 wt % MF resin) before and after impregnation. The higher initial viscosity and larger viscosity increase of 8-30MF resulted from the higher expected polymerization rate of MF resin at lower pH. Nevertheless, the viscosity of the 8-30MF formulation was still considered low and not a problem for achieving suitable impregnation of the wood boards.^31,32^ Simultaneously, the upper pH of 8-30MF is also limited due to the lower water solubility of BA at higher pH. BA converts into borax under alkali conditions, and borax has a lower water solubility than BA. Thus, pH 7.3–7.6 was determined as the optimized condition for the 8-30MF formulation preparation.

**Table 1:** The pH and Viscosity of the Formulations before and after Impregnation

formulation	0-30MF	8-30MF
pH	8.4–8.6	7.3–7.6
viscosity before impregnation (cP)	3.48	4.94
viscosity after impregnation (cP)	3.88	5.97

The curing of the impregnated MF formulation within the wood structure has been found to increase the risk of crack formation in the treated material due to the volume shrinkage of the step-growth polymerization of the MF resin.^33^ Therefore, relative humidity and temperature were carefully controlled during the curing and drying process. The curing temperature was set above the glass transition temperature of the wood at 60 °C, aiding softening of the moisture-saturated wood and reducing the crack formation.^34,35^ The high relative humidity during curing also helped reduce the formation of cracks.^33^

### Bulking Coefficient and Weight Percentage Gain

**Figure 1:**
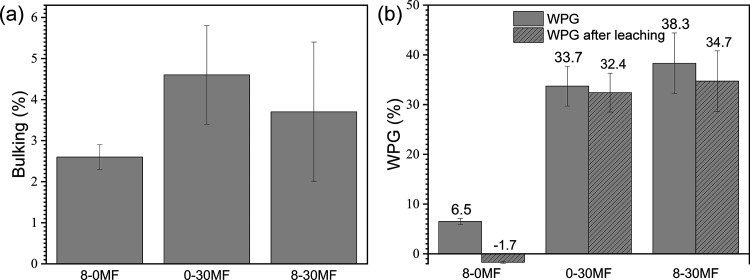
Values of (a) bulking coefficient (BC) and (b) weight percentage gain (WPG) of 8-0MF, 0-30MF, and 8-30MF specimens before and after water leaching.

The bulking coefficient (BC) was calculated by measuring the macroscopic dimension of the specimens according to eq 2 to assess the penetration of the formulation into the cell wall.^36^ The penetration of the formulation would occupy some volume in the cell wall and increase the macroscopic dimensions of dried boards. No BC suggests that the formulation was mostly deposited in voids such as the lumina, and thus, the macroscopic dimension would not be changed. Figure 1a shows that 8-0MF (8 wt % FRs), 0-30MF, and 8-30MF specimens gave increased BCs of 2.5, 4.5, and 3.5%, respectively. The results imply that both FRs and MF resin penetrated the cell wall during impregnation and remained there after curing. FRs seem to easily penetrate the swollen cell wall as they could be smaller than microvoids formed in the swollen cell wall. The penetration of MF resin is also favored as its relatively low molecular weights make it easier to diffuse into the cell wall as previously reported.^32^

Weight percentage gain (WPG) data showed the amount of loaded chemicals within the wood structure after the treatments (Figure 1b). WPG depends on various parameters such as the solid content and viscosity of the formulation, impregnation procedures, wood species and tissues, etc. The 8-0MF specimen had only 6.5% WPG as the solid content in the formulation was low (8 wt % of the FRs). The modified wood containing MF resin, 0-30MF and 8-30MF specimens, had higher WPG due to the higher solid content and relatively low viscosity of the MF resin in these formulations. Thus, considerable amounts of the MF resin penetrated and remained in the material after the treatments. Comparing the WPG difference before and after leaching with water according to EN 84 can be used to examine the water resistance of the modified wood (Figure 1b), with a smaller WPG difference indicating better water resistance of the loaded chemicals. Figure 1b shows that the increased WPG of the 8-0MF specimen was completely lost after water leaching due to the hygroscopicity of the FRs. The negative value of WPG may be due to the wood being degraded by hot water, leading to an increase in extractives during leaching that was performed according to EN 84.^37^ The standard deviations of 0-30MF and 8-30 MF specimens were relatively high resulting in low statistical differences before and after water leaching. Nevertheless, the WPG difference of 0-30MF and 8-30MF specimens was quite similar compared to the 8-0MF specimen, which is due to the hydrophobic cured MF resin providing good water resistance.^33^

**Figure 2:**
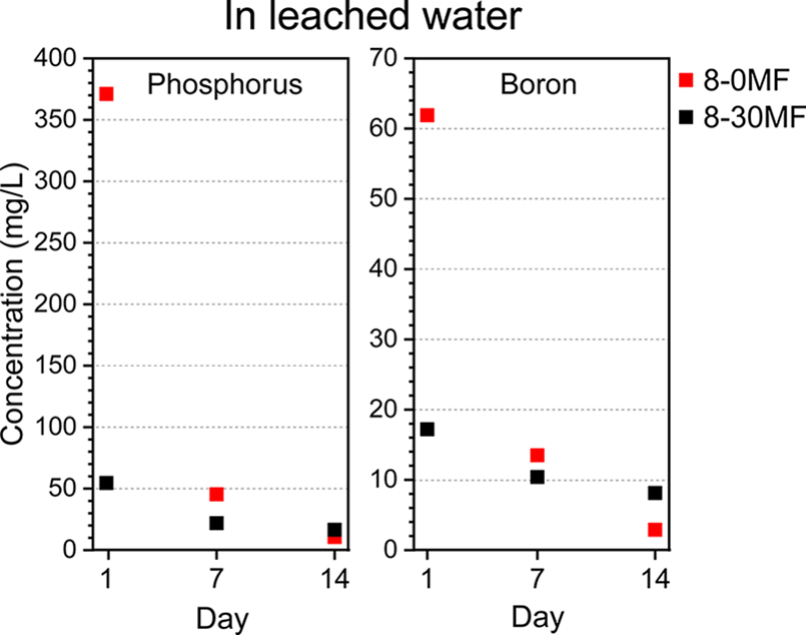
Results of ICP-AES and ICP-SFMS determination of P and B in leached water solutions.

Inductively coupled plasma-atomic emission spectroscopy (ICP-AES) and inductively coupled plasma-sector field mass spectrometry (ICP-SFMS) analyses were used to identify the concentration of phosphorus and boron elements in the collected water extracts from leaching by EN 84. The phosphorus element was mostly derived from GUP, while the boron element was mostly attributed to BA as the unmodified Scots pine had a negligible amount of these elements (Table S1). The concentrations of phosphorus and boron elements from water leaching collected at day 1, 7, and 14 of 8-0MF and 8-30MF specimens are shown in Figure 2. The incorporation of MF resin significantly reduced the release of FRs into water, with a reduction of seven times of the phosphorus element and three times of the boron element concentrations in day 1 water. FRs in the 8-30MF specimen were released slowly during the 14-day leaching period, while FRs in the 8-0MF specimen were dramatically released at day 1 water and resulted in low FRs in the day 14 water. ICP results indicate that the incorporation of polymers into the wood matrix slowed down the release of hydrophilic additives.

### Morphology, Elemental Composition, and Chemical Functionalities

**Figure 3:**
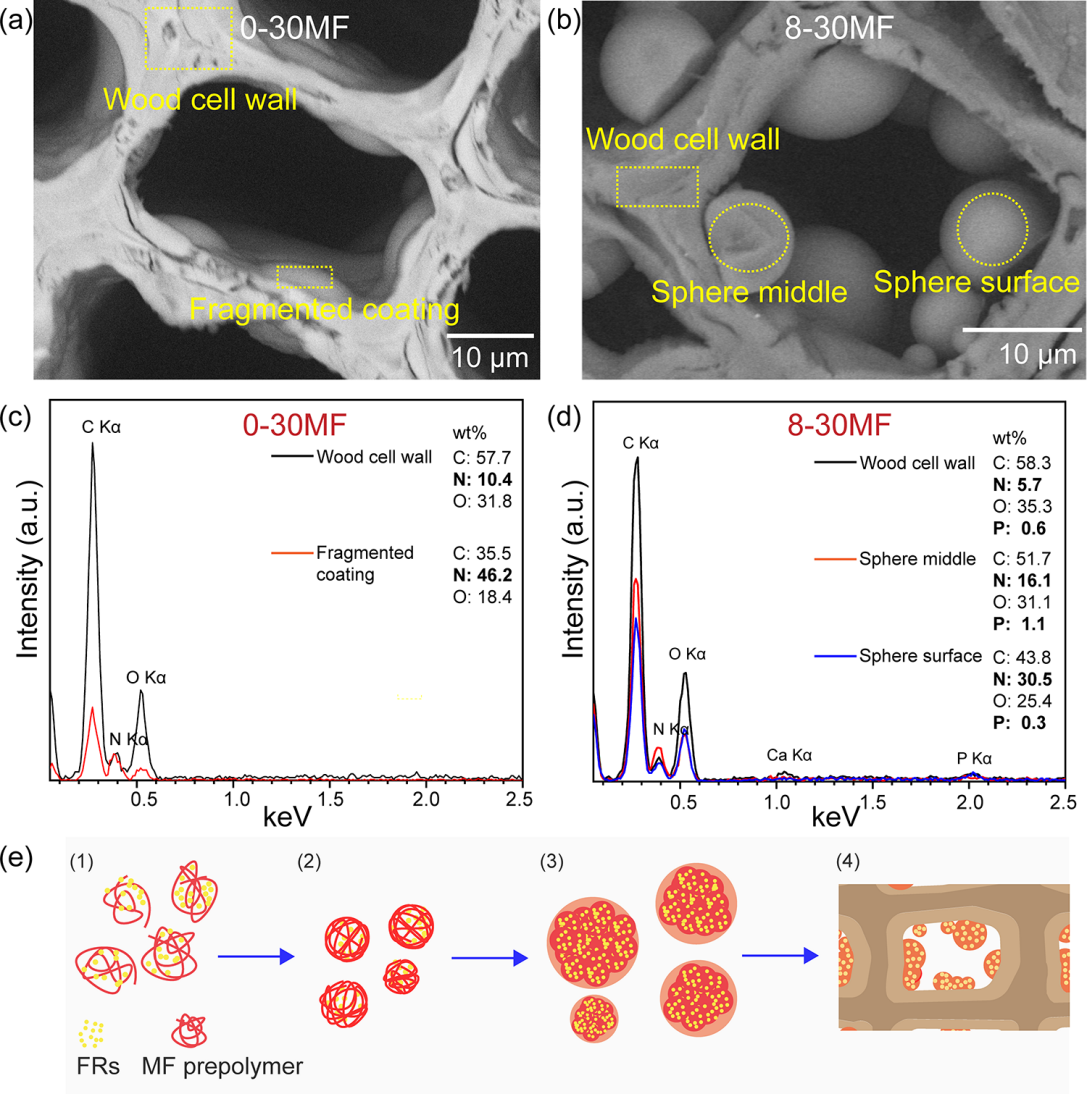
(a–d) Cross-sectional SEM images and the corresponding EDX spectra of 0-30MF and 8-30MF specimens and (e) schematic illustration of the FR doping mechanism. Adapted with permission from ref (30). Copyright 2014 The Royal Society of Chemistry.

The natural cellular structure of the unmodified Scots pine sapwood observed by SEM is presented in Figure S1a. SEM images showed that there were no significant changes observed in the morphology of wood modified with FRs (Figure S1b). The 0-30MF specimen exhibited a slightly different morphology (Figure 3a), and homogeneous coverage of the internal sides of the cell lumina was observed. Some places also showed the fragmented coating close to internal layers of the cell wall (Figure 3a), and this was attributed to the strain in the convex meniscus surface of the MF prepolymer droplets induced by the curing process. On the contrary, the 8-30MF specimen showed a different morphology, with numerous microspheres formed inside the lumina (Figure 3b and Figure S2a).

To investigate the distribution of elements within the modified wood structure as well as validate the mechanism of microsphere formation, energy-dispersive X-ray spectrometry (EDX) was performed for the unmodified and modified wood. The EDX analysis confirmed that the elements C and O were detected for the unmodified Scots pine (Figure S1c), which were attributed to their presence in the main components of wood: cellulose, hemicelluloses, and lignin. The EDX spectrum of the 8-0MF specimen showed elements such as C, N, O, Al, and P (Figures S1d and S3). The wt % ratios of these elements varied and were found to be place-dependent. The elements P and N detected in the cell walls were attributed to GUP (additional EDX elemental mappings are presented in Figure S3), while Al was from the sample holder. This further confirmed the penetration of FRs into cell walls and agrees with results when an increase in the BC of the specimens was observed. The detection of Ca in several samples was assigned to the inorganic elemental composition of the natural wood.^38^ The EDX spectra of the 0-30MF specimen, collected from the regions of the cell wall and coating from the internal side of the cell wall (Figure 3a,c), showed the presence of the N element indicating the penetration of the MF resin into the cell walls. This further suggests that diffusion of the MF prepolymer into the cell walls followed by in situ polymerization may result in cell wall reinforcement.^32^ Previous reported studies show that the diffusion of the prepolymer after curing may permanently bulk the cell walls and thus increase the dimensional stability of treated wood.^39^ The MF fragmented coatings were also observed in the bordered pits (Figure S2b), which resulted in completely filled-in pits/voids. The EDX spectra of the formed microspheres within the cell lumina of the 8-30MF specimen suggested that the microspheres consisted of mostly MF resin with P encapsulated within the formed spheres (Figure 3b,d). SEM/EDX analysis showed that a higher concentration of P was observed in the middle of the microspheres. It has also been mentioned that the N content in the cell walls for the polymerized composites (0-30MF and 8-30MF specimens) was higher compared to that detected in wood modified with single FRs, i.e., the 8-0MF specimen (Figure S3 shows EDX spectra). This relevantly high difference in concentration of N can be attributed to the actual chemical composition of the analyzed material, i.e., the cell wall contains additional N content from MF resin. Furthermore, the increase in N within the polymerized material indicates a good affinity between the MF precursor and GUP, further suggesting this to be a promising route for entrapping inorganic compounds within the organic matrix. The formation of these microspheres might proceed via an organic sol–gel doping mechanism, which was reported by Wu et al.^30^Figure 3e shows a schematic representation of the microsphere formation mechanism and its stages: (1) FRs and the MF prepolymer are initially dissolved in water; (2) water is removed from the saturated solution, so the saturated FRs act as nuclei and are further wrapped by the continuous polymerizing MF resin; (3) continuously polymerizing MF resin aggregates and grows in dimension; (4) consequently, FR-doped MF resin spheres with a microscale diameter are observed in the lumina.

**Figure 4:**
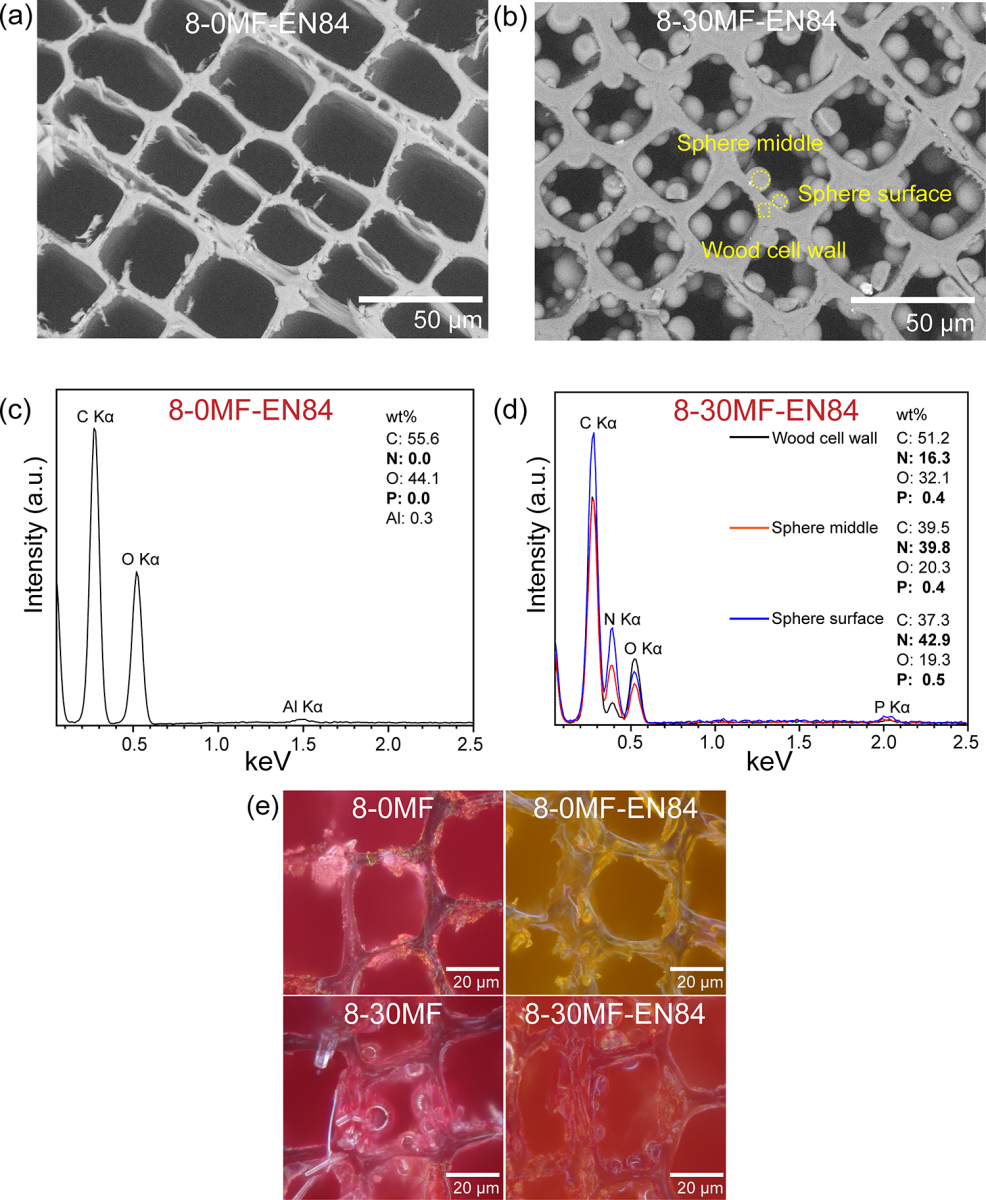
(a–d) Cross-sectional SEM images and the corresponding EDX spectra of 8-0MF and 8-30MF specimens after water leaching and (e) optical microscopy images of BA reagent-colored 8-0MF and 8-30MF wood before and after water leaching.

Moreover, to validate the encapsulation of the FRs within the polymer matrix that was used to modify Scots pine, the water-leached samples were studied by EDX. The water-leached 8-0MF specimen showed no P in the EDX spectrum, indicating that GUP has been washed out from the modified wood by water leaching (Figure 4c). On the contrary, the EDX spectrum of the water-leached 8-30MF specimen showed P, so confirming its encapsulation within the microspheres (Figure 4d). The N signals of 8-30MF-EN84 were noted to be higher than those of 8-30MF. This could be due to the loss of BA during water leaching. As a molecule of BA contains three oxygen atoms, the loss of oxygen will result in the ratio changes of all elements noted in the EDX spectra.

Due to the difficulty in detecting the B element in EDX, the use of optical microscopy with the curcumin–BA coloring reagent was undertaken to visualize the BA distribution in cross sections isolated from the modified wood.^40,41^ The wood coloring method, based on color difference due to the reaction of curcumin with BA, is described in the Supporting Information. Optical microscopy images (Figure 4e) suggested the removal of BA in the 8-0MF specimen since the yellow color after water leaching was very similar to that of the unmodified Scots pine (Figure S4a). On the contrary, the 8-30MF specimen showed a reddish color with a partially yellow tint after water leaching, which indicated that BA was only partially removed (Figure 4e). Additionally, the reddish color of the microspheres in the 8-30MF specimen indicated that BA was present and thereby also involved the organic sol–gel doping mechanism in MF microspheres. The MF resin had no effect on the BA coloring reagent since the unmodified Scots pine and the 0-30MF specimen exhibited similar colors (Figure S4b). These results are in agreement with ICP analysis, where low P and B contents were measured in water leached from the 8-30MF wood (Figure 2).

**Figure 5:**
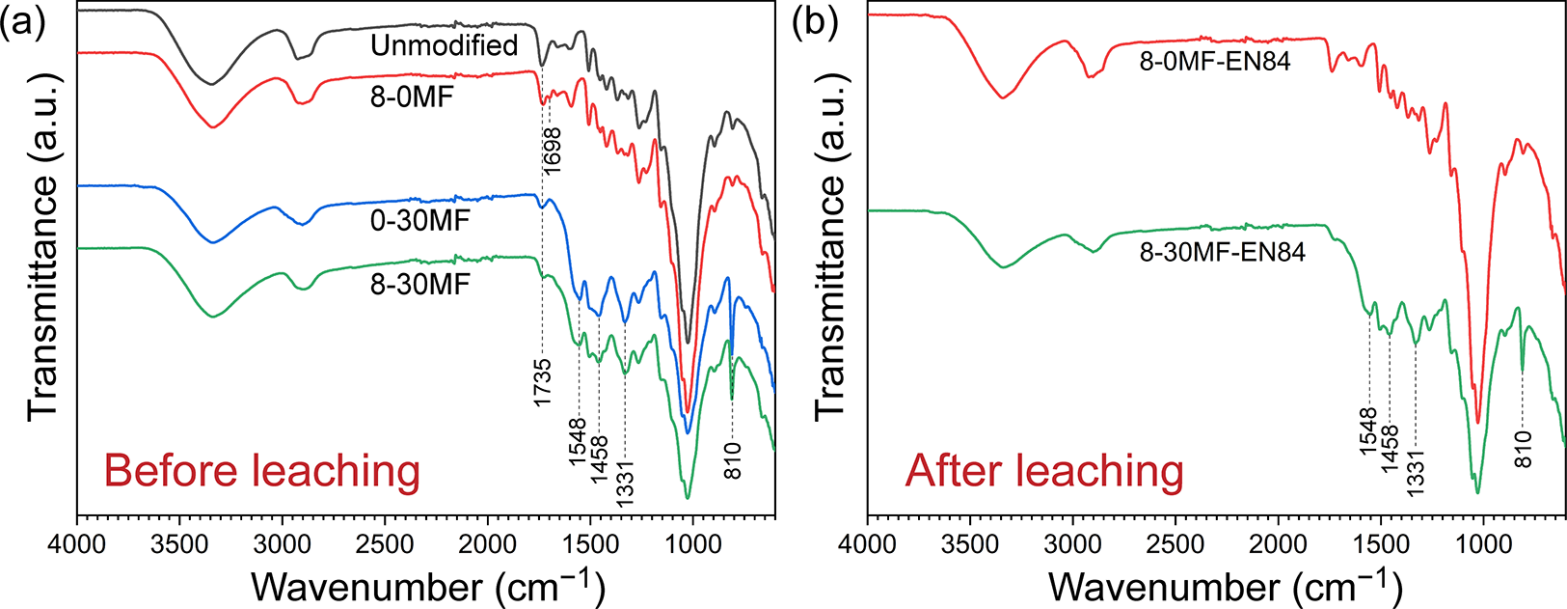
FTIR spectra of (a) unmodified Scots pine, 8-0MF, 0-30MF, and 8-30MF specimens and (b) 8-0MF and 8-30MF specimens after water leaching.

The chemical functionalities of the unmodified Scots pine, 8-0MF, 0-30MF, and 8-30MF specimens were analyzed by FTIR, shown in Figure 5a. The band assignments of characteristic absorption bands are given in Table 2.

**Table 2:** Assignment of FTIR Bands of Unmodified Scots Pine, 8-0MF, 0-30MF, and 8-30MF Specimens

wavenumber (cm^–1^)	assignment
3600–3000	O–H stretching in polysaccharides^47^ and lignin^48^
2980–2820	C–H asymmetric and symmetric stretching of methoxyl groups and methyl and methylene groups in wood^47^ and C–H asymmetric stretching in methylene bridges between triazine rings in MF resin^45^
1735	C=O stretching in unconjugated ketones, carbonyls, esters, or O–acetyl groups in hemicellulose^48−50^
a1698	C=O stretching^42,43^
1652	C=O stretching in conjugated ketones^48^
1635	H–O–H deformation of adsorbed water^48^
1605 and 1508	C=C aromatic skeletal vibrations in lignin^48^
b1548	C=N triazine ring vibrations^45^
1462	C–H asymmetric bending in lignin^48^
b1458	C–H bending in methylene bridges between triazine rings^45^
1452	C–H asymmetric bending in lignin^47^ and O–H in-plane bending^51^
1422	C–H aromatic skeletal vibrations^48^
1370	C–H bending in polysaccharides and lignin^48,50^ and O–H bending in phenol^48^
1338	C–H in-plane bending in cellulose^47^ and C–O stretching in lignin^47^
b1331	C–H in-plane bending in methylene bridges between triazine rings^45^
1315	CH_2_ wagging in cellulose^47^
1262	C–O stretching in lignin^48^
1234	C–O stretching and O–H in-plane bending in polysaccharides^49^
1152	C–O–C stretching in polysaccharides^49^
1020–1050	C–H in-plane bending in lignin,^48^ C–O stretching in primary alcohol,^48^ and C–O–C stretching in polysaccharides^51^
896	C–H out-of-plane bending in polysaccharides^51^ and lignin^48^
b810	triazine ring from melamine or melamine formaldehyde resin^45^
808	C–H out-of-plane bending in positions 2, 5, and 6 of coniferyl alcohol in lignin^48^

a The new peak attributed from GUP.

b The new peak attributed from cured MF resin.

The FR-modified wood specimen (8-0MF) showed an absorption band at 1698 cm^–1^, which was assigned to the C=O functional groups of GUP.^42,43^ The bands at 3187, 1407, and 1190 cm^–1^ related to BA did not appear in the FTIR analysis of the 8-0MF specimen and might be due to the low amount of BA present in the treated wood. FTIR spectra of GUP and BA are presented in Figure S5. The MF resin-modified wood specimen (0-30MF) showed both a reduction in original bands and new peak formation. The intensity of C=O related to hemicellulose at 1735 cm^–1^ was reduced, probably due to the cleavage of the O–acetyl bond in hemicellulose, as previously found during wet heat curing conditions.^44^ The cleavage of O–acetyl bonds may produce acetic acid, which can accelerate the curing of MF resin, with formation of stable methylene bridges. The methylene bridge could be attributed to the absorption bands at 1458 and 1331 cm^–1^ as had been presented earlier.^45^ Those absorption bands were found in the 0-30MF specimen as well as absorption bands at 1548 and 810 cm^–1^ assigned to the triazine ring vibrations of melamine. The FR with MF resin-modified wood specimen (8-30MF) revealed a similar spectrum to 0-30MF. Analysis of the 8-30MF specimen spectrum showed the bands assigned to the MF resin contribution at 1548, 1458, 1331, and 810 cm^–1^. However, the absorption band related to GUP at 1698 cm^–1^ was not distinguished as the MF resin might overlap the absorption band of GUP.

The FTIR spectra of 8-0MF and 8-30MF specimens after water leaching are shown in Figure 5b. The 8-0MF specimen showed no absorption band at 1698 cm^–1^ due to the removal of GUP as a result of water leaching. On the contrary, the spectrum of the 8-30MF specimen was similar to the nonleached spectrum. The absorption bands at 1548, 1458, 1331, and 810 cm^–1^ assigned to MF resin were still present due to the hydrophobic nature and higher molecular weight of the cured MF resin.^46^

### Thermal Behavior

**Figure 6:**
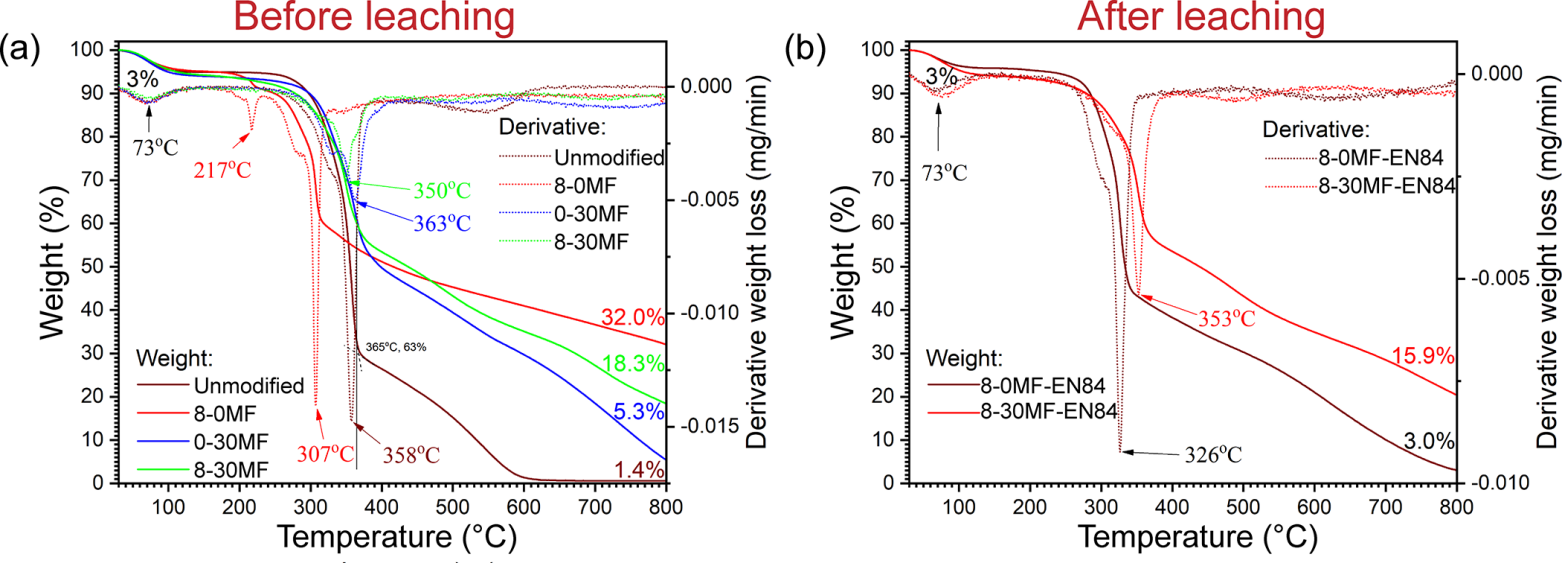
TGA and DTG curves of (a) unmodified Scots pine, 8-0MF, 0-30MF, and 8-30MF specimens and (b) 8-0MF-EN84 and 8-30MF-EN84 specimens.

Thermal gravimetric analysis (TGA) and differential thermal gravimetric (DTG) curves of the unmodified Scots pine, 8-0MF, 0-30MF, and 8-30MF specimens are presented in Figure 6a. For the unmodified Scots pine, the 3% weight loss in the first stage from 30 to 105 °C was assigned to the removal of absorbed moisture within the wood structure. The second stage from 250 to 380 °C with maximum decomposition temperature at 358 °C was mostly attributed to the rapid pyrolysis stage of the wood components hemicellulose and cellulose.^52^ Lignin starts to decompose slowly at this stage. Hemicellulose decomposes from 225 to 325 °C. Cellulose is more thermally stable than hemicellulose with decomposition temperature from 280 to 370 °C. Dehydration, decarboxylation, and decarbonylation processes occur at this stage, producing small molecules such as H_2_O, CO_2_, and CO.^52^ The third stage from 380 to 600 °C is mainly related to the decomposition of lignin with the cleavage of the linkages between monolignols and vaporization of monomeric phenols. Lignin is more thermally stable than hemicellulose and cellulose due to its aromatic structure; the decomposition starts slowly at 200 °C and finishes at 600 °C.^52^ Above 600 °C weight loss is related to the further decomposition of the carbonized and aromatic structure.^53^ The remaining carbon-rich residue at 800 °C constituted about 1.4% of its initial weight.

The FR-modified wood specimen (8-0MF) had an extra degradation stage at 180 °C (peak at 217 °C in the DTG curve), which was related to the degradation of FRs. The nitrogen-rich guanyl-urea segment in GUP releases low-combustible or incombustible gases, such as NH_3_ and CO_2_.^8^ Simultaneously, the phosphate segment in GUP undergoes dehydration with formation of a pyrophosphate bond.^54^ The pyrophosphate will further condense as polyphosphate, with the polyphosphate acting as protective glassy layers preventing further degradation of the wood. The thermal degradation of BA occurs instantaneously at 180 °C.^8^ The dehydration of BA is accompanied with release of water and formation of B_2_O_3_ protective glassy layers. The main decomposition temperature of the 8-0MF specimen was shifted to a lower temperature of 307 °C (Figure 6a). The acidity of GUP and BA promoted the thermal degradation of wood, leading to increased char formation at elevated temperature.^8^ Consequently, the carbon-rich residue of the 8-0MF specimen at 800 °C was 32.0% of its initial weight, which was much higher than that of the unmodified Scots pine (Figure 6a).

The MF resin-modified wood specimen (0-30MF) showed slightly faster thermal degradation than unmodified Scots pine from 120 to 310 °C (Figure 6a). This was probably due to the degradation of MF resin. Such degradation with formation of small amounts of formaldehyde, methanol, and amines has been reported.^55^ The main decomposition stage, between 310 and 380 °C, involved the degradation of the wood as well as the cleavage of the side group of the MF resin. Small molecules such as formaldehyde, methanol, NH_3_, CO_2_, or simple amines were released in this stage.^55^ From a temperature of 380 to 650 °C, the MF resin deaminates to form condensed cyameluric (heptazine) structures,^55^ which provides better thermal stability and results in a higher residue weight and a higher main decomposition temperature than the unmodified Scots pine (TGA and DTG curves of pure MF resin are presented in Figure S6). At a temperature above 650 °C, the condensed cyameluric structure is gradually degraded into volatile products including CO_2_, HCN, and CO.^55^ The residue of the 0-30MF specimen at 800 °C was found to be 5.3% of its initial weight.

The FR/MF resin-modified wood specimen (8-30MF) did not reveal any obvious thermal degradation stage at 180 °C as the 8-0MF specimen. This might be due to the FRs encapsulated within the MF resin microspheres. The MF resin microsphere could efficiently prevent the early thermal degradation of FRs.^19,56^ It was also evident that the 8-30MF specimen had a similar TGA curve to 0-30MF below 380 °C. At a temperature above 380 °C, the degradation of MF resin started, and the doped FR was released from the MF microspheres. Consequently, the released FRs promoted char formation and resulted in the 8-30MF specimen having a higher residue weight than the 0-30MF specimen at 800 °C, at 18.3% of its initial weight. Specimen 8-30MF had a lower residue weight than 8-0MF at 800 °C due to the release of volatile products from degraded MF resin during TGA.^57^

TGA and DTG curves of the 8-0MF-EN84 and 8-30MF-EN84 specimens, presented in Figure 6b, show that the 8-0MF-EN84 specimen had no extra thermal degradation step at 210 °C, which was due to the removal of FRs during water leaching. It was also noticed that the carbon-rich residue at 800 °C was 3.0%, higher than that of the unmodified Scots pine, which may be due to the removal of wood components by the acidic FRs during water leaching (Figure 1).^58^ The TGA result was consistent with the results of WPG, ICP, and SEM/EDX; the FRs were not stable within the wood structure without the incorporation of the resin matrix. In contrast, the 8-30MF-EN84 specimen showed similar TGA and DTG curves to those of the 8-30MF specimen (Figure 6a,b), both having maximum decomposition temperatures around 350 °C. The 8-30MF-EN84 specimen had a slightly lower amount (16% compared to the 18% for the 8-30MF specimen) of the carbon-rich residue at 800 °C due to the partial removal of FRs and MF resin during water leaching.

### Flammability

**Figure 7:**
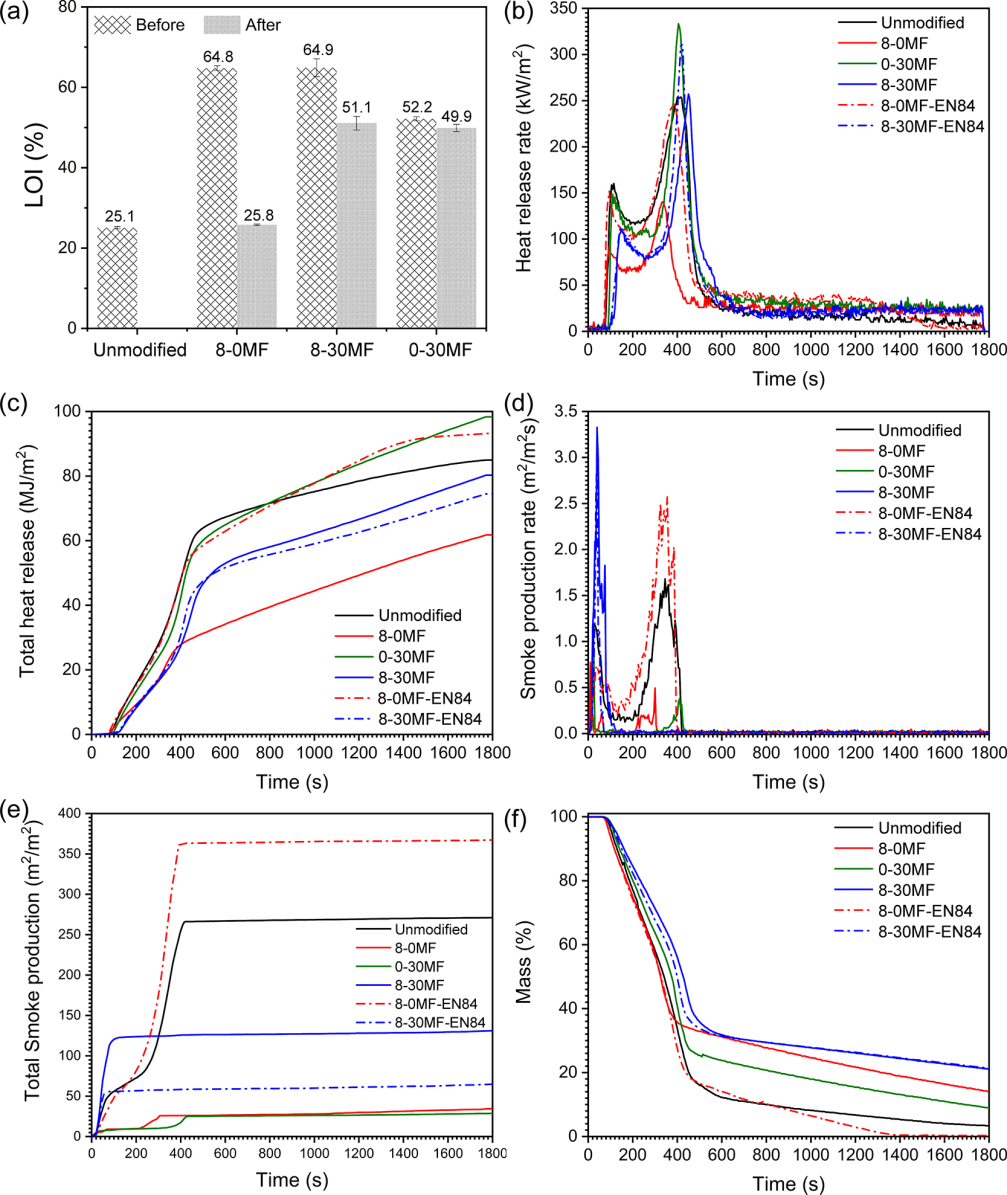
(a) LOI results of the unmodified Scots pine, 8-0MF, 0-30MF, 8-30MF, and 8-0MF, and 8-30MF specimens after water leaching and (b–f) cone calorimeter results of the heat release rate (HRR), total heat release (THR), smoke production rate (SPR), total smoke production (TSP), and mass loss of the unmodified Scots pine, 8-0MF, 0-30MF, 8-30MF, and 8-0MF, and 8-30MF specimens after water leaching.

Limiting oxygen index (LOI) analysis was used as a screening fire test by measuring the minimum O_2_ concentration that supported ignition, with a higher LOI value indicating that the specimen required higher O_2_ concentration to be ignited. The LOI of the unmodified Scots pine required 25.1% O_2_ (Figure 7a). The wood modified with FRs (the 8-0MF specimen) had a significantly higher LOI (64.8%). Nevertheless, FRs themselves did not permanently bond within the wood structure, and thus, the LOI of water-leached 8-0MF dropped to 25.8%, which is a similar value to that of the unmodified Scots pine. The MF resin and FR-modified wood (the 8-30MF specimen) showed a similar LOI to that of the 8-0MF specimen, with a value of 64.9%. Water-leached 8-30MF (8-30MF-EN84) had a similar LOI value to 0-30MF, about 50%. The previous studies reported that the LOI value of MF resin-modified Scots pine sapwood was around 40%.^59,60^ The LOI value of pure MF resin described in ISO 4589-2:2017 was also around 40%.^61^ The 25% higher LOI value of MF resin-modified wood in this study might be due to the MF resin recipe differing from that of various manufacturers. Consequently, the remaining FRs in 8-30MF-EN84 might not truly reflect the fire retardancy during the LOI test.

As a result of the LOI tests, cone calorimeter testing was undertaken to further investigate the fire retardancy of the modified wood, shown in Figure 7b–f and Table 3. The test provides an insight into the fire behavior developed by presenting various important parameters, including the heat release rate (HRR), total heat release (THR), smoke production rate (SPR), total smoke production (TSP), mass loss, and time to ignition (TTI). The HRR is considered the most important parameter to evaluate the growth and spread of fire.^62^ The first peak in the HRR curve (pHRR_I_) corresponds to the oxidation of the wood with the release of heat. As the burning continues, the protective char layers form and lower the HRR, shown as the valley before the second peak (pHRR_II_). The cause of the second peak is specimen overheating (during the cone calorimeter test) and subsequent release of flammable volatile products not only from the exposed side of the specimen but also from the opposite side of the specimen. This hypothesis is supported by the fact that the second peak is always observed just before complete burning of the specimen. The following decrease in the HRR corresponds to nonflaming burning (glowing) after the volatile products are consumed.^2^ The FR-modified wood specimen (8-0MF) showed that the FRs significantly suppressed the heat release of the modified wood because GUP/BA promoted the char formation and formed protective glassy layers for inhibiting the heat release.^2^ The melamine resin-modified wood specimen (0-30MF) exhibited a higher pHRR_II_ and THR than the unmodified Scots pine, which is attributed to the exothermic reaction of the breakage of the methylene bridge in the cured resin.^45^ This implied that the incorporated MF resin would promote the fire hazard of the modified wood. By contrast, with the introduction of FRs into the MF resin-modified wood (8-30MF), heat release was suppressed, and both pHRR_I_ and pHRR_II_ were shifted to a longer exposure time. This indicated that the fire growth rate of the treated wood was lowered by the incorporation of FRs to the MF resin treatment.

**Table 3:** Time to Ignition (TTI), Maximum Average Rate of Heat Emission (MARHE), Total Heat Release (THR), and Total Smoke Production (TSP) of the Unmodified Scots Pine, 8-0MF, 0-30MF, 8-30MF, and 8-0MF-EN84 and 8-30MF-EN84 Specimens after Water Leaching (± Standard Deviation)

specimen	unmodified Scots pine	0-30MF	8-0MF	8-0MF-EN84	8-30MF	8-30MF-EN84
TTI (s)	86 ± 7	96 ± 1	81 ± 1	77 ± 2	128 ± 4	121 ± 15
MARHE (kW/m^2^)	133 ± 5	123 ± 1	70 ± 3	125 ± 8	93 ± 1	96 ± 5
THR (MJ/m^2^)	85 ± 15	98 ± 1	62 ± 1	93 ± 7	80 ± 1	74 ± 3
TSP (m^2^/m^2^)	271 ± 17	29 ± 19	35 ± 5	367 ± 24	131 ± 53	65 ± 11

Apart from the heat release, the production of smoke (e.g., particulate matter or volatile organic compounds) is also an important parameter in evaluating the fire hazard of the material. The smoke production rate (SPR) and total smoke production (TSP) are presented in Figure 7d,e, and it could be seen that FR or MF resin treatment can significantly suppress the smoke production; the effect of FRs is due to its expected fire retardancy,^2^ while that of the MF resin is due to the more complete combustion of the modified wood into a yield of CO_2_ and N_2_ instead of smoke.^25^ The FR/MF resin-modified wood specimen (8-30MF) showed a higher SPR and TSP than the 8-0MF or 0-30MF specimens, which might be due to FRs accelerating the decomposition of MF resin, producing more volatiles at the beginning of the test. Nevertheless, 8-30MF specimens still reduced about 50% of TSP compared to the unmodified Scots pine (Table 3).

The mass residue of the combusted FRs or MF resin-modified wood was enhanced compared to the unmodified Scots pine (Figure 7f) due to the mentioned char promotion and protective layer formation properties of the used chemicals.^2,25^ The FR/MF resin-modified wood (the 8-30MF specimen) had an even higher mass residue than the solely FRs or MF resin-modified wood specimens. This might be due to the temperature of the cone calorimeter test with a heat flux of 50 kW/m^2^ corresponding to about 600 °C;^63^ the thermal degradation temperature of the condensed cyameluric structure from MF resin is about 650 °C.^57^ Thus, the degradation of the 8-30MF specimen was slower than the observation in TGA and resulted in a higher mass residue than the 8-0MF specimen.

The improvement of the MF resin on the water resistance of the FR-modified wood can be seen by comparing the 8-30MF and 8-30MF-EN84 specimens. The slight increase in the pHRR_II_ after water leaching could be due to the partial loss of FRs. The decreased total smoke production (TSP) could be explained by the 8-30MF-EN84 specimen having a shorter time to ignition (TTI). The short TTI tends to have a lower smoke release amount because a significant amount of smoke has been released before ignition (in the time interval between thermal loading of the sample and flame ignition). As a reference, the leached FR-modified wood specimen (8-0MF-EN84) released more smoke than the unmodified Scots pine during the first 400 s of testing (Figure 7f). On the other hand, the mass losses of both specimens were almost equal during the first 400 s of the test (Figure 7f). The probable cause is that the removed hydrophilic FR specimens increased the combustion behavior (increase total heat release) and smoke production (Figure 7c, e, and f).

Time to ignition (TTI) and maximum average rate of heat emission (MARHE) results are shown in Table 3. TTI is defined as how quick the combustion occurs when the material is subjected to external heat flux. A higher TTI value indicated that longer time was required for the material to reach the pyrolysis temperature^62^ and a decreasing fire hazard of a material. 8-30MF and 8-30MF-EN84 specimens had superior TTIs among the modified woods. The result indicated that the FRs with MF resin treatment could prolong the time of the modified Scots pine to be ignited. MARHE is defined as the peak value selection of the averaged HRR divided by the corresponding time interval. The mathematic expression of MARHE was originally reported by Marquis et al.^64^ MARHE is used as an important criterion for comparing different materials’ fire hazards within the European standard EN 45545-2:2013.^65^ A lower MARHE value indicated a lower fire hazard of the material. The 8-0MF specimen had the best MARHE among the specimens, about 70 kW/m^2^, while the 8-30MF specimen increased the value to about 90 kW/m^2^. From the data, we suggested that FRs with MF resin treatment resulted in a slightly increased fire hazard. Any such decrease in original fire retardancy is more than compensated for by the superior fire retardancy of the water-leached specimen (8-30MF-EN84) due to the excellent water resistance of the cured MF resin.

## Conclusions

A leach-resistant fire-retardant treated wood was successfully developed by doping hydrophilic fire-retardant GUP/BA to MF resin microspheres. The results of ICP, SEM/EDX, and TGA all showed evidence of incorporation of melamine formaldehyde (MF) into the wood structure to enhance the water leaching resistance of hydrophilic fire retardants. Furthermore, SEM/EDX confirmed the formation of MF microspheres with the doping of GUP in the GUP/BA/MF resin-treated specimen. Additionally, EDX also confirmed that GUP was still present in the microspheres of the leached specimen. Flammability tests carried out using a cone calorimeter and the LOI showed high fire stability of the modified wood, even after the accelerated artificial aging test (i.e., excessive leaching with water). To the authors’ knowledge, this is the first research study dealing with the incorporation of MF resin microspheres and hydrophilic FRs to achieve highly leach-resistant, fire-retardant modified wood. This provides new insight into using conventional hydrophilic FRs for exterior-use, fire-retardant modified wood.

In summary, this new approach is applicable to nonrefractive wood materials, and it would be interesting, for future works, for this to be applied and adopted for other types of wood products such as thermally modified timber and acetylated wood.

## Experimental Section

### Materials

Scots pine (*Pinus sylvestris* L.) sapwood, knot- and crack-free, was obtained from a sawmill in Skellefteå, Sweden. A pine-heartwood indicator was used to ensure no presence of heartwood in the wood material. The indicator was prepared by mixing equal volumes of *o*-anisidine solution (0.5 g of *o*-anisidine with 2 mL of 37% HCl dissolved in 100 mL of deionized (DI) water, VWR Puranity TU 3) and NaNO_2_ solution (10 g of NaNO_2_ dissolved in 100 mL of DI water). After spraying of the indicator, the presence of any heartwood section would have turned the cross-cut end a red color, while the sapwood section would remain yellow.

Specimens of dimensions 100 × 10 × 100 mm (tangential (*T*) × radial (*R*) × longitudinal (*L*)) and 10 × 10 × 150 mm (*T* × *R* × *L*) were conditioned at 20 °C and 65% relative humidity (RH) to reach an equilibrium moisture content (EMC) of around 12% before measuring the mass (*m*_12_) and volume (*v*_12_). The density of the conditioned wood was 500 ± 50 kg/m^3^.

Purities of 98% of guanyl-urea phosphate (C_2_H_9_N_4_O_5_P, GUP) and 99% of *o*-anisidine were obtained from Fisher Scientific, Sweden. ACS-grade boric acid (H_3_BO_3_, BA), analysis-grade sodium hydroxide (NaOH) pellets, and fuming ACS-grade of 37% hydrochloric acid (HCl) were purchased from Merck, Germany. Melamine formaldehyde (MF) resin powder was provided by Dynea AS, Norway. The molecular weight was analyzed by size exclusion chromatography as presented in Supporting Information, Figure S7. MF resin powder was dissolved entirely in DI water before use. All chemicals were used as received without additional purification.

### Preparation of the Fire Retardant Formulations

Three different formulations were prepared: (1) a water solution containing 8 wt % FR (the mass ratio of GUP/BA is 7:3), denoted as 8-0MF, (2) a water solution containing 30 wt % MF resin, denoted as 0-30MF, and (3) a water solution containing 8 wt % FR and 30 wt % MF resin, denoted as 8-30MF.

To prepare the 8-30MF formulation, the FRs were dissolved completely in DI water prior to slowly adding to the MF resin solution under continuous stirring. The formulation was then adjusted to pH 7.3–7.6 (the pH value was measured by VWR Dosatest pH test strips pH 6.0–10.0) by the addition of NaOH (10 M) to minimize any immediate condensation.

### Preparation of the Modified Wood

The formulations were impregnated into the specimens (10 replicates) following a vacuum-pressure technique. The specimens were fully immersed in the formulations prior to impregnation for 1 h at 20 mbar followed by 3 h at 15 bar, establishing a full cell impregnation of the specimens. Then, the excess formulation on the surface of the specimens was removed by tissue paper before curing and drying in a kiln drier (Valutec AB, Sweden). The kiln drier curing and drying procedure was performed in three steps: (1) 75 °C and 99% RH for 24 h, (2) RH linearly reduced from 99 to 65% over 20 h while keeping at 75 °C, and (3) 75 °C and 65% RH for 4 h. The mass (*m*_1_) and volume (*v*_1_) of the cured specimens were measured for calculating the weight percentage gain (WPG) and bulking effect (BC). WPG and BC were determined using eqs 1 and 2, respectively.
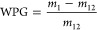
1
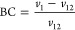
2

### Accelerated Aging Test

The test was carried out on five replicates following European standard EN 84:1997,^66^ i.e., all specimens were fully immersed in five times more volume of DI water in a polypropylene container before applying 20 min of vacuum at a reduced pressure of 20 mbar. Water was replaced ten times during the 14-day leaching period. Water was changed at the first and last day of immersion. The changing of water was eight times in between at intervals of not less than one day and not more than three days. The leached specimens were then dried following the same procedure as the curing steps for preventing the crack formation on the surface. The dried mass after leaching was measured as *m*_2_. WPG after the EN 84 European standard was calculated according to eq 3.
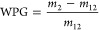
3

### Characterization

A rotary viscometer (AMETEK Brookfield DV2T-LV) equipped with a ULA spindle was utilized to measure the viscosity of the fire retardant formulations. The measurements were performed at 20 °C, and the rotation speed of the spindle was fixed at 40 rpm. Size exclusion chromatography (SEC) was performed on a TOSOH EcoSEC HLC-8320 GPC system equipped with an EcoSEC RI detector and three PSS PFG 5 μm columns (Microguard, 100 and 300 Å), using dimethylformamide as a solvent with 0.01 M LiCl as the mobile phase at 50 °C under a flow rate of 0.2 mL min^–1^ to analyze the molecular weight and distribution of MF resin powder. Toluene was used as an internal standard. Fourier transform infrared spectroscopy (FTIR) was performed using a PerkinElmer FTIR Frontier spectrometer equipped with a UATR Diamond/ZnSe ATR (Single Reflection) over the wavenumber range of 4000–600 cm^–1^ with 8 scans at a resolution of 4 cm^–1^. The surface of the specimens was cut out by a microtome blade for the FTIR analysis. Thermal gravimetric analysis (TGA) was performed using a PerkinElmer TGA 4000, where 4 ± 1 mg of each specimen was loaded in an alumina crucible and heated at a rate of 10 °C min^–1^ from 30 to 800 °C under a N_2_ flow rate of 20 mL min^–1^. The surface of the specimen was cut out by a microtome blade for TGA. At least three replicates were performed for each specimen. The first-order derivative of the TGA curve (DTG curve) was smoothed by a 20-point-smooth algorithm through the Metter Toledo STARe Evaluation V16.20 software. The morphology and elemental composition of the specimens were characterized by scanning electron microscopy (SEM) using a Jeol JSM-IT300LV equipped with an energy-dispersive X-ray spectrometer (EDX). The spectrometer was controlled through the Oxford Instrument ZAtec V3.1 software. The cutting of specimens was performed using a microtome blade. The specimens without coating were examined using secondary electrons, and the electron beam acceleration voltage was set at 15 kV under low-vacuum mode at 100 Pa, using a scanning time of 160 s for the EDX mapping. Each specimen has three replicates of the analysis. An optical microscope Olympus DSX-1000 capable of 40× magnification was also used to obtain the morphology of the specimens. Inductively coupled plasma (ICP) was carried out by ALS Scandinavia AB Luleå to determine the boron and phosphorus concentration in leached water following EN 84:1997. Water was first acidified with HNO_3_ to reach 1% HNO_3_ in water before analyzing. The boron concentration was analyzed by ICP-atomic emission spectroscopy (ICP-AES) using an Agilent ICP-OES 725. The phosphorus concentration was analyzed by ICP-sector field mass spectrometry (ICP-SFMS) using a Finnigan MAT Element 1. Flammability tests were carried out on the limiting oxygen index (LOI) and the cone calorimeter. The LOI (Fire Testing Technology Ltd., UK) test was conducted by following ISO 4589-2:2017 on five replicates with a dimension of 10 × 10 × 150 mm (*T* × *R* × *L*).^61^ The cone calorimeter (dual cone calorimeter, Fire Testing Technology Ltd., UK) test was performed according to ISO 5660-1:2015 under a heat flux of 50 kW/m^2^ on five replicates with a dimension of 100 × 10 × 100 mm (*T* × *R* × *L*).^67^

## Supporting Information Available

ICP analysis of concentration of phosphorus and boron of the unmodified wood; cross-sectional SEM images and EDX elemental mapping of the unmodified Scots pine and 8-0MF specimen; radial-section SEM images of 8-30MF and 0-30MF specimens; three replicates of 8-0MF EDX spectra; optical microscopy cross-sectional images of the BA reagent-colored unmodified Scots pine and 0-30MF specimen; FTIR spectra of GUP and BA; TGA and DTG curves of MF resin; molecular weight distribution of MF resin powder; curcumin-BA coloring method preparation (PDF)

## ABBREVIATIONS

AES: atomic emission spectroscopy
BA: boric acid
BC: bulking coefficient
cP: centipoise
DI: deionized
DTG: derivative thermal gravimetry
EDX: energy-dispersive X-ray spectrometer
EMC: equilibrium moisture content
FRs: fire-retardant additives
FTIR: Fourier transform infrared spectroscopy
GUP: guanyl-urea phosphate
HCl: hydrochloric acid
HRR: heat release rate
ICP: inductively coupled plasma
*L*: longitudinal
LOI: limiting oxygen index
MC: moisture content
MF: melamine formaldehyde
NaOH: sodium hydroxide
NH_3_: ammonia
*R*: radial
RH: relative humidity
SEC: size exclusion chromatography
SEM: scanning electron microscopy
SFMS: sector field mass spectrometry
SPR: smoke production rate
*T*: tangential
TGA: thermal gravimetric analysis
THR: total heat release
TSP: total smoke production
TTI: time to ignition
WPG: weight percentage gain
