# Supplementary material for: Approaching Highly Leaching-Resistant Fire-Retardant Wood by In Situ Polymerization with Melamine Formaldehyde Resin
Source: ACS Omega. 2021 May 6;6(19):12733–45. doi: 10.1021/acsomega.1c01044 (PMC8154219; doi:10.1021/acsomega.1c01044)
Supplement: Supplementary file 1 — ao1c01044_si_001.pdf [file ao1c01044_si_001.pdf]

Supporting information

Approaching high leaching-resistant fire-retardant wood by *in-situ* polymerization with melamine formaldehyde resin

Chia-feng Lin[*a], *Olov Karlsson*[a], *Jozef Martinka*[b], *Peter Rantuch*[b], *Edita Garskaite*[a], *George I. Mantanis*[c], *Dennis Jones*[a, d] *and Dick Sandberg*[a, d]

[a]Wood Science and Engineering, Department of Engineering Sciences and Mathematics, Luleå University of Technology, Forskargatan 1, SE-931 77 Skellefteå, Sweden

[b]Faculty of Materials Science and Technology, Slovak University of Technology, Vazovova 5, SK-811 07 Bratislava, Slovakia

[c]Lab of Wood Science and Technology, University of Thessaly, Griva 11, GR-43100, Karditsa, Greece

[d]Department of Wood Processing and Biomaterials, Faculty of Forestry and Wood Sciences, Czech University of Life Sciences Prague, Kamýcká 1176, Praha 6 - Suchdol, CZ-16521, Czech Republic

*corresponding authors

Table S1. ICP analysis of concentration of phosphorous and boron of the unmodified wood

Figure S1. Cross-sectioned SEM images and EDX elemental mapping of the unmodified Scots pine and 8-0MF specimen

Figure S2. Radial-sectioned SEM images of 8-30MF and 0-30MF specimens

Figure S3. Three replicates of 8-0MF EDX spectra

Figure S4. Optical microscopy cross-sectional images of BA reagent colored of unmodified Scots pine and 0-30MF specimen

Figure S5. FTIR spectra of GUP and BA

Figure S6. TGA and DTG curves of MF resin

Figure S7. Molecular weight distribution of MF resin powder

Curcumin-BA coloring method preparation.

Table S1. Inductively coupled plasma (ICP) analysis of concentration of phosphorous and boron of the unmodified wood (leached water collected after 1, 7, and 14 days).

Day	Phosphorous concentration (mg/L)	Boron concentration (mg/L)
1	0.095	0.868
7	0.042	0.766
14	<0.04	0.637

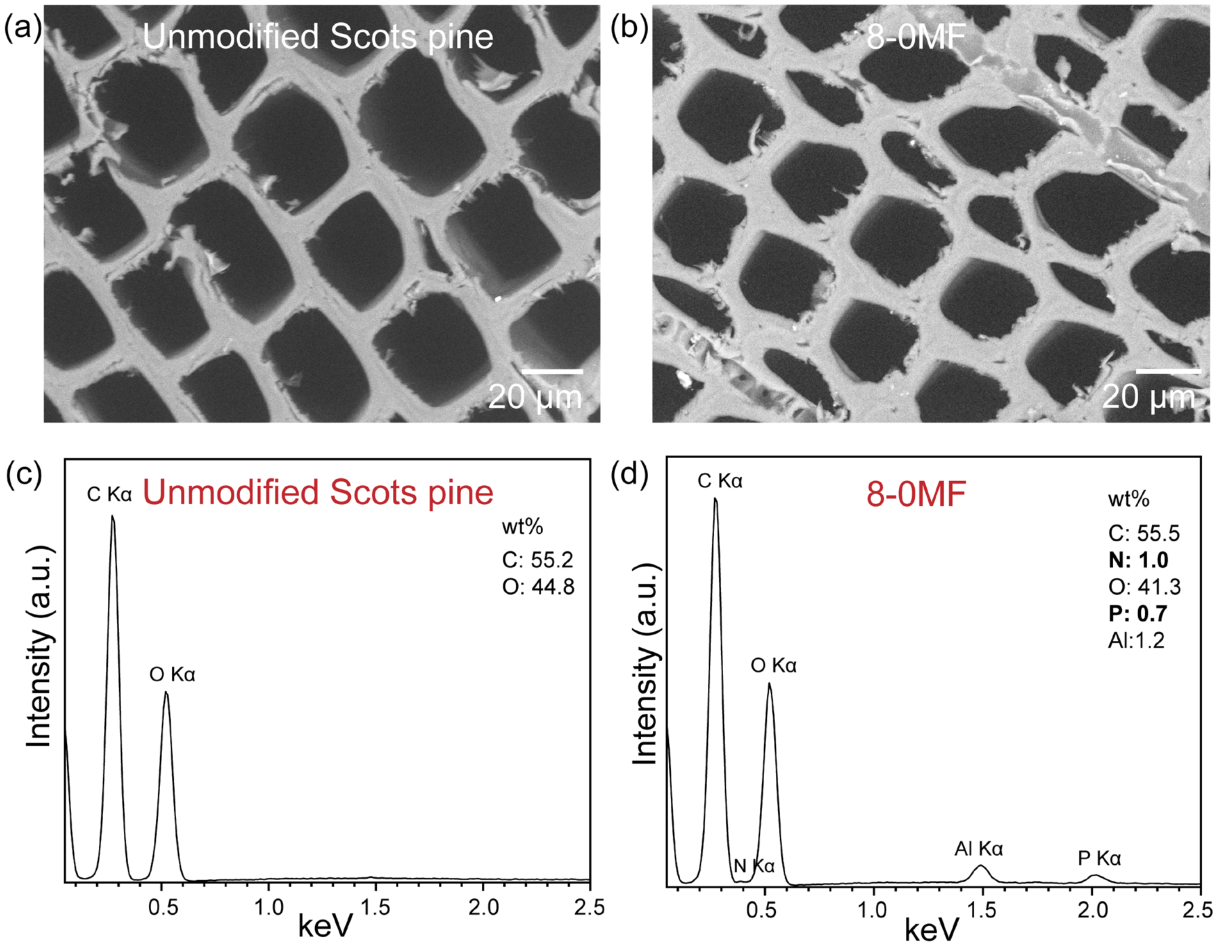

Figure S1. Cross-sectioned SEM images (a) unmodified Scots pine, (b) 8-0MF specimens with its corresponded EDX spectra in (c) and (d), respectively.

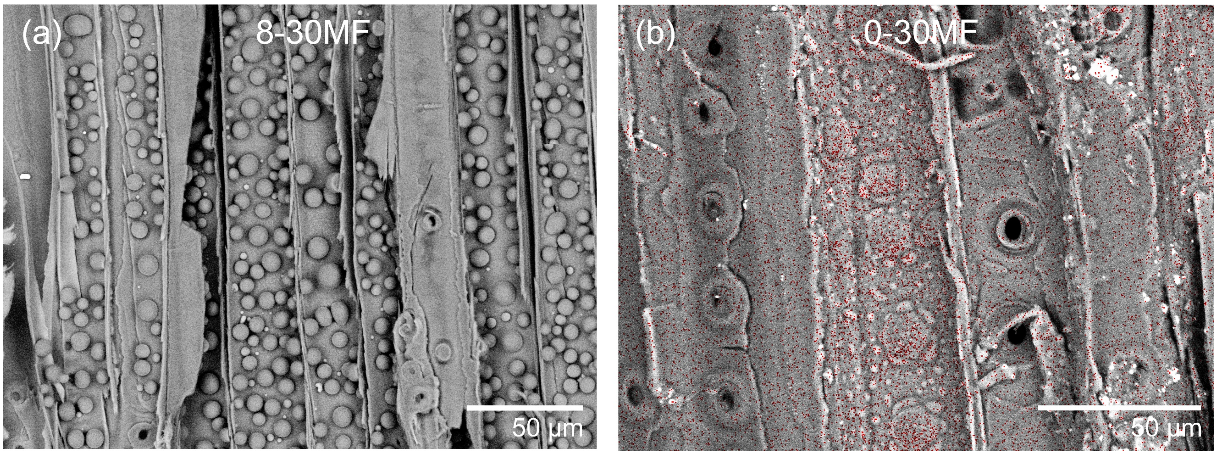

Figure S2. Radial-sectioned SEM images of (a) 8-30MF specimen, and (b) 0-30MF specimen with elemental N designated by red color dots.

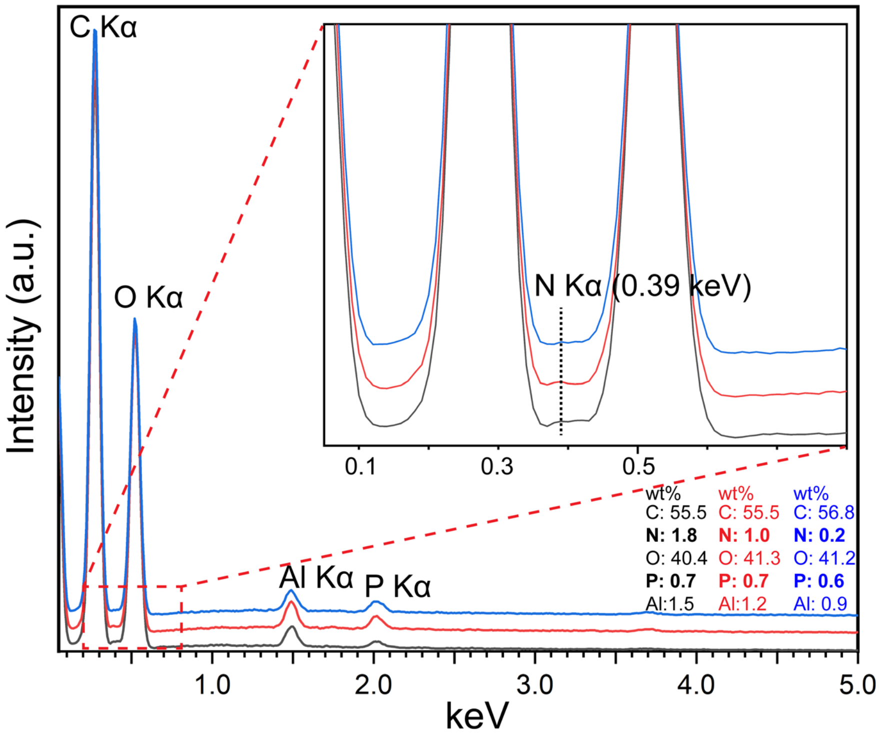

Figure S3. Three replicates of 8-0MF EDX spectra.

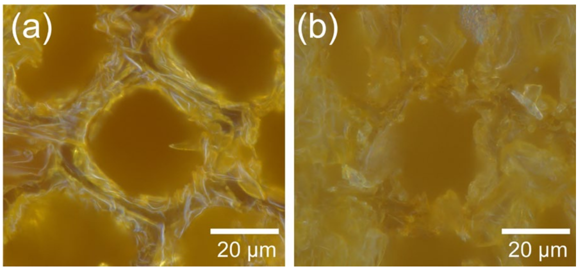

Figure S4. Optical microscopy cross-sectional images of BA reagent colored of (a) unmodified Scots pine, and (b) 0-30MF specimen.

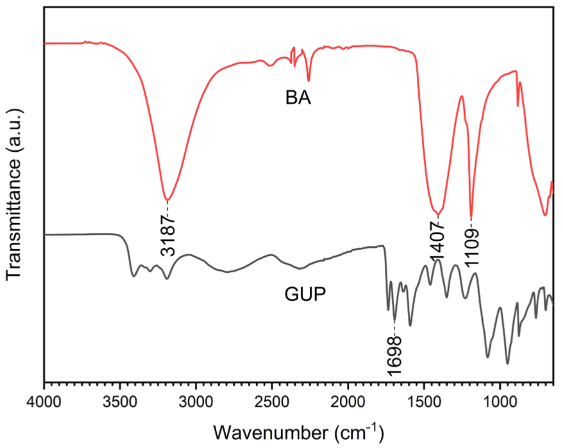

Figure S5. FTIR spectra of GUP and BA.

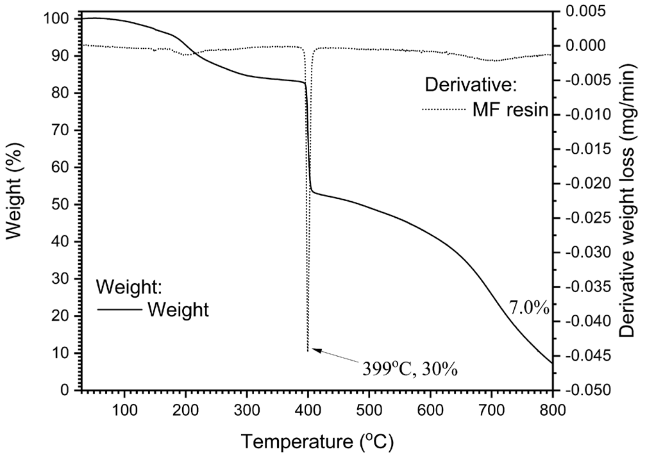

Figure S6. TGA and DTG curves of the MF resin. The MF resin was cured under the same curing condition as the 0-30MF specimen in the kiln drier.

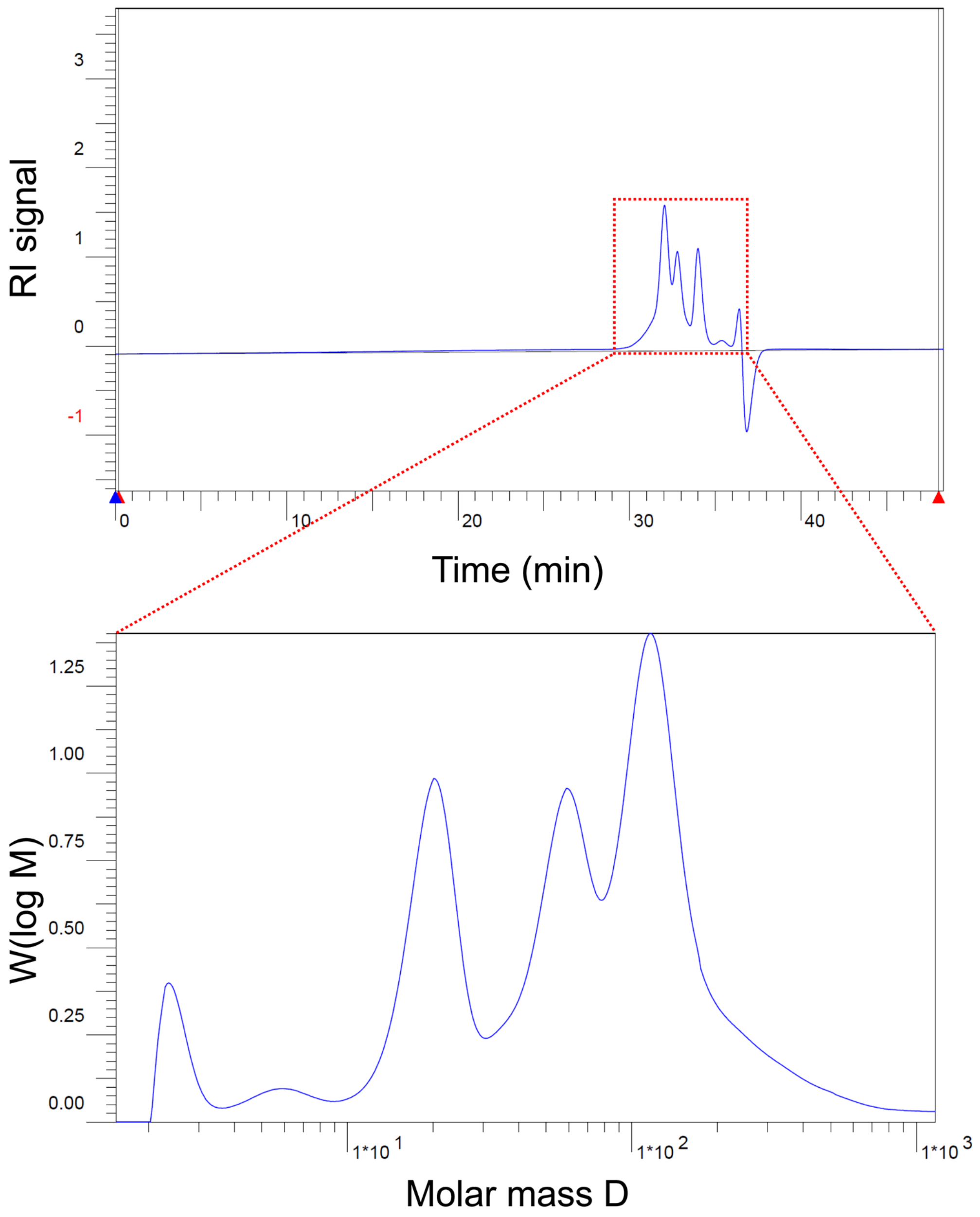

Figure S7. Molecular weight distribution of MF resin powder, analyzed by size exclusion chromatography (SEC).

Curcumin-BA coloring method preparation.

BA coloring method follows dropping 10 μL of solutions A and B respectively on the specimens. The solution A was prepared by dissolving 0.5 g of curcumin in 10 ml of ethanol. Solution B was prepared by mixing 2 ml of 37% HCl, 1.3 g of salicylic acid in 8 ml of ethanol.

10 μL of solution A was initially dropped on the cross-section of the specimens. 10 μL of solution B was dropped on the same spot after solution A had fully dried out. The dried specimen was then observed under the optical microscopy to investigate the color changes.
